# High-risk twin pregnancy: case report of an adolescent patient with cystic fibrosis and systemic lupus erythematosus

**DOI:** 10.1186/s13256-022-03399-3

**Published:** 2022-05-31

**Authors:** Paula Baptista Sanseverino, Anneliese Hoffmann, Sandra Machado, Mariana Farias, Marcus Silva Michels, Maria Teresa Vieira Sanseverino, Paulo José Cauduro Marostica

**Affiliations:** 1 Universidade Federal do Rio Grande do Sul-PPG Saúde da Criança e do Adolescente, Ramiro Barcelos 2400 /sala 220, Atanásio Belmonte 515/502, Porto Alegre, RS 90520550 Brazil; 2 Hospital de Clínicas de Porto Alegre, Ramiro Barcelos 2350 /sala 1131, Porto Alegre, Brazil; 3 Hospital de Clínicas de Porto Alegre, Ramiro Barcelos 2350, Porto Alegre, Brazil; 4 Universidade Federal do Rio Grande do Sul-Serviço de Genética HCPA, Ramiro Barcelos 2350, Porto Alegre, Brazil; 5 Hospital de Clínicas de Porto Alegre-Serviço de Genetica, Ramiro Barcelos 2350, Porto Alegre, Brazil

**Keywords:** Cystic fibrosis, Systemic lupus erythematosus, Adolescent pregnancy, Multiple pregnancy

## Abstract

**Background:**

We present the first case to our knowledge of a spontaneous twin pregnancy in a 16-year-old Caucasian patient with cystic fibrosis and systemic lupus erythematosus. Cystic fibrosis is one of the most common autosomal recessive genetic disorders and primarily affects the respiratory and digestive systems. Systemic lupus erythematosus is a chronic inflammatory disease of unknown cause that affects nearly every organ. Patients with cystic fibrosis or systemic lupus erythematosus are progressively having longer life expectancy and better quality of life, which has led a greater number of female patients reporting the desire to become mothers.

**Case presentation:**

We present a case of a Caucasian 16-year-old pregnant with twins being treated for both cystic fibrosis and systemic lupus erythematosus. She has two *CFTR* mutations: p.F508del and 1812_1G>A. In the second trimester, she was admitted for possible preterm labor, which was successfully stopped. The patient’s nutritional status worsened, and she had a pulmonary exacerbation as well as a flare of systemic lupus erythematosus. At the 28th gestational week, she presented with a massive hemoptysis episode. The cesarean delivery had no complications, and there were no serious immediate postpartum complications.

**Discussion and conclusions:**

While adolescent pregnancies in and of themselves are considered high risk for both the young mothers and their children, they are further complicated when the mother has two chronic diseases and a twin pregnancy. We achieved positive results using a multidisciplinary approach; however, the risks involved were so high that major efforts are to be taken by our medical community to prevent unplanned pregnancies in all patients with cystic fibrosis, especially when a serious comorbidity like the one in this case is present.

## Background

The latest Brazilian cystic fibrosis (CF) registry (2016) reported that 3.6% of adult female patients with cystic fibrosis become pregnant. In the same period, the Cystic Fibrosis Foundation Patient Registry (CFFPR) reported a rate of approximately four live births per 100 women who are of reproductive age with CF in the USA. Pregnancy in patients with CF requires continuous monitoring and treatment to maintain maternal pulmonary function and the added nutritional needs of gestation. Furthermore, it requires avoiding, when possible, the use of teratogenic medications in the treatment of pulmonary infections.

Systemic lupus erythematosus (SLE) is a chronic inflammatory disease of unknown cause that affects almost every organ. Cystic fibrosis is also an inflammatory disease, and studies suggest that the presence of inflammation in CF airways is independent of previous infection [1]. As with CF, a better understanding of the pathophysiology of SLE and the involvement of a multidisciplinary team over the last few decades led to an improvement of feto-maternal outcomes. Pregnancy can be associated with flares in patients with SLE [2].

Twin and teenage pregnancy are both independent risk factors for adverse pregnancy outcomes [3].

We present the first case to our knowledge of a spontaneous twin pregnancy in a 16-year-old Caucasian patient with cystic fibrosis and SLE.

## Case presentation

The 16-year-old Caucasian patient presented here has CF and is followed regularly at our CF clinic. She has two identified *CFTR* mutations: p.F508del and 1812_1G>A. She has also been seen by our hospital’s rheumatology team since she was diagnosed with SLE with hematologic activity in September 2016. On that occasion, she presented with thrombocytopenia, antinuclear antibody (ANA) 1:160 with speckled pattern, low c4 measure, Coombs test with agglutination intensity 3 out of 4 and intermittent arthralgia.

During a hospital admission for a pulmonary exacerbation in August 2017, she was treated with intravenous antibiotics and methotrexate was momentarily discontinued. Prior to resuming methotrexate, a known teratogenic drug, a pregnancy blood test (β-human chorionic gonadotropin) was conducted.

A pregnancy was then detected, and she was placed on prenatal follow-up with the obstetrics team. A first-trimester ultrasonography showed that she had a monochorionic and diamniotic twin pregnancy. The patient was also treated for syphilis during her first trimester [Venereal Disease Research Laboratory (VDRL) 1:4 on 18 September 2017]. In the second trimester, she was admitted for possible preterm labor, which was successfully stopped. However, the patient’s nutritional status worsened, and she had a pulmonary exacerbation. Intravenous antibiotics (ceftazidime and oxacillin) were then initiated on the basis of her sputum cultures and safety profile concerning teratogenic risks. She also presented a SLE flare with thrombocytopenia and was treated with corticosteroids with a good response.

During pregnancy, a worsening in her lung function was observed. Her lung function prior to gestation showed an obstructive ventilatory pattern with a forced vital capacity (FVC) 110% and first second of forced expiratory volume (FEV1) 76% of predicted.

**Fig. 1:**
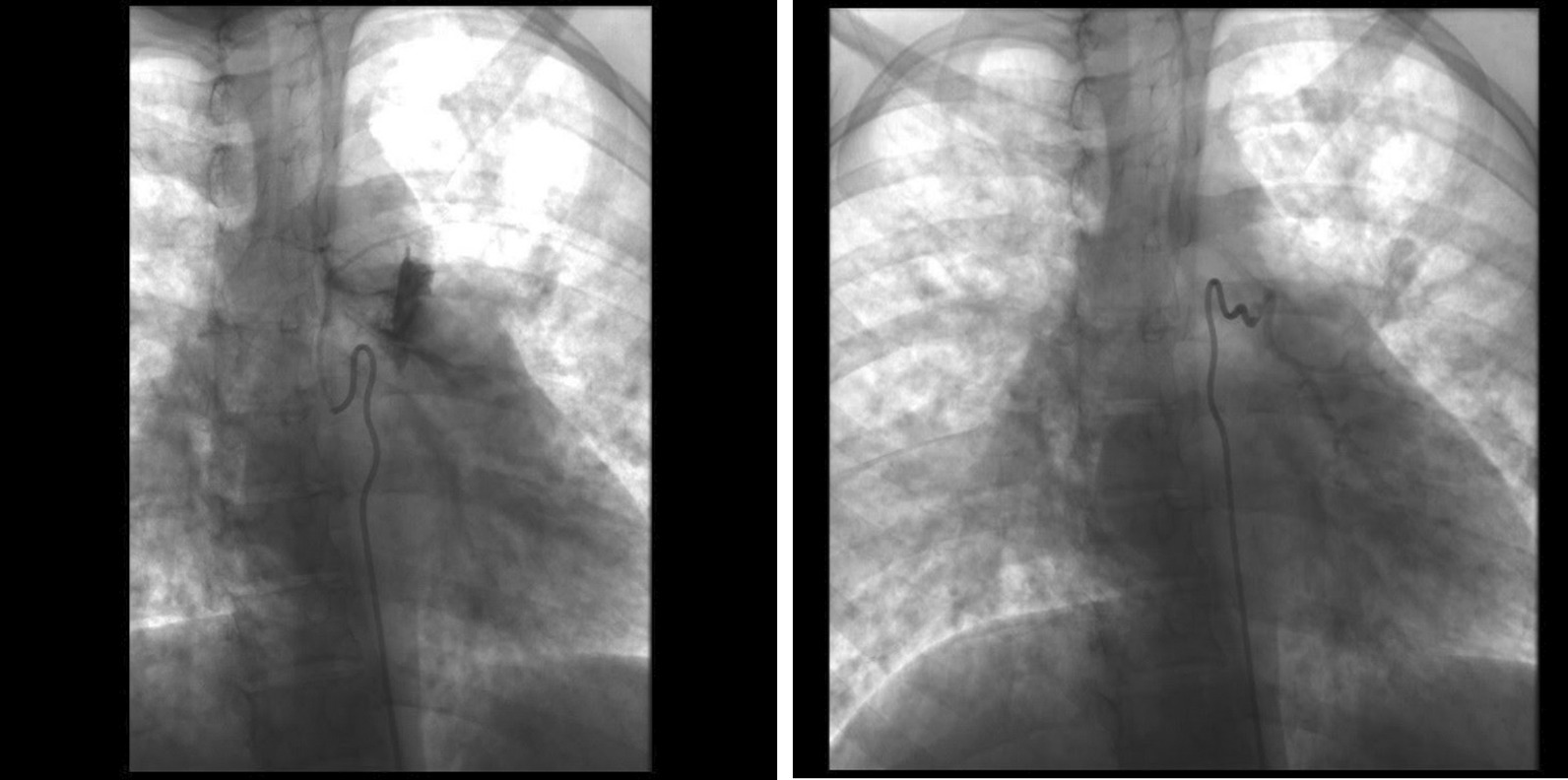
Embolization of right bronchial artery with abnormal contrast impregnation of pulmonary parenchyma

Since admission during her second trimester, she struggled to gain weight. To improve her nutritional status, additional enteral feeding was started, and the patient had slow but progressive weight gain. At the 28th gestational week, she presented a massive hemoptysis episode. A bronchial artery embolization was then indicated. However, considering the radiation exposure involved in the procedure and the associated risk for the patient and the fetuses, a cesarean section was conducted beforehand (Fig. 1).

The cesarean delivery in late January 2018 had no complications. The first-born baby weighed 880 g with an Apgar score of 1 and 8, and the second baby weighed 980 g with an Apgar score of 3 and 7, both being admitted to neonatal intensive care unit (ICU). They needed mechanical ventilation for at least their first month of life. The firstborn was soon diagnosed with ventricular septal defect and was managed with diuretics while awaiting surgical correction. The second baby needed supplemental oxygen but was slowly and successfully weaned off.

Our patient had no serious immediate postpartum complications. Both babies were discharged from the pediatric ward and are doing well at home so far. Our patient, however, has had several exacerbations since her delivery, mostly due to her lack of adherence to CF treatment. She also struggled with depression after birth and is now medicated and doing better in this regard.

## Discussion and conclusions

In the past few years, the life expectancy and quality of life of patients with cystic fibrosis have improved. This has led to increasing numbers of reports of female patients wishing to become mothers. In our pediatric service, this was the first pregnant adolescent patient. As discussed by Jessup *et al.*, the growing number of parents with cystic fibrosis has implications not only for them, who already have the burden of CF care, but also for their children’s risk of premature death [4]. Tonelli *et al.* suggested that psychological support for mothers with CF and their children ought to be provided to decrease the incidence of depression, guilt, self-criticism, and lack of self-confidence [5]. Our patient suffered from postpartum depression, including suicidal ideation, and responded well to psychiatric support. Our patient’s treatment adherence was not optimal before or during her pregnancy. It was clear that, especially in the first months, her CF treatment was neglected. Authors from a large single-center study in the USA recommended aggressive management for all pregnant women with cystic fibrosis, including the use of parenteral nutrition to maintain adequate weight gain [6]. The nutritional status of this patient was another challenge, since she was losing weight during the second trimester of pregnancy, and as a result she remained hospitalized to receive adequate treatment. Financial costs and patient burden are often high during pregnancy in patients with cystic fibrosis, including the high number of prenatal visits (mean 12), days on intravenous antibiotics (mean 30 days), and total hospitalization days, both pre- and postpartum (mean 25 days) [6].

During pregnancy in patients with CF, lung function can worsen because of increased abdominal growth, minute ventilation, and oxygen demand [7]. Gestational diabetes mellitus and presence of cardiovascular risk factors can also worsen the prognosis of patients with cystic fibrosis and systemic lupus erythematosus [8, 9].

The treatment of pulmonary exacerbations in this case was also a challenge encompassing the selection of adequate safe antibiotic regimens for pregnant women in addition to the management of hemoptysis. Even though there is a growing number of studies evaluating the safety of these drugs during pregnancy, this topic is still rarely discussed in the literature. In this case, adequate treatment based on the sputum bacteriological profile was only possible after labor [10]. The interruption of her pregnancy at 28 weeks due to a massive hemoptysis was also part of this challenge.

Regarding the outcomes in pregnancies of patients with CF, Gilljam *et al.*, in a single-center study from Toronto, found no long-term detrimental effects in women with cystic fibrosis who had children. Goss *et al.*, using the large national US Cystic Fibrosis Foundation Registry, confirmed and expanded upon this finding [11, 12]. Another American paper, by Schechter *et al.* in 2013, showed that neither pregnancy nor motherhood appeared to accelerate disease progression, although pregnancy and motherhood does lead to more illness-related visits, pulmonary exacerbations, and a decrease in some domains of quality of life. They speculated that these differences may be due to the impact of the physical and emotional challenges of early motherhood on disease self-management [13], which is in accordance with our patient´s status today.

Increased mucus clogging and lung injury in CF, some studies showed, are associated with neutrophil cytotoxins, extracellular DNA, and neutrophil extracellular traps (NETs). Dysregulated NET formation or clearance has been associated with CF and chronic inflammatory and autoimmune disorders [14].

Considering the rheumatologic aspects in a patient with SLE, the maternal fetal outcomes have improved in the last decades. Khan *et al.* highlight the importance of a multidisciplinary team to minimize complications [2]. The flare rates during pregnancy of women with SLE vary in the literature from 25% to 65% [15–19]. Khan *et al.* showed that the majority of SLE flares were mild to moderate. The patient presented with thrombocytopenia since her SLE diagnosis, and this persisted during pregnancy, as the flares were well managed solely with corticosteroids. Other complications related to SLE during pregnancy, such as preeclampsia, maternal death, and spontaneous abortion, did not occur in this case [20, 21].

In a Brazilian cohort, 14 out of 24 young pregnant SLE women were still followed by a pediatrician. In this same study, 21% of the patients had disease activity during their pregnancy, similar to the patient reported here. They found that less than 10% had mild flares after the onset of pregnancy and responded to temporary increase in corticosteroid dosage [21].

Sexual behavior of teenagers with chronic diseases does not differ from that of other teenagers. It is important to provide advice on contraception and prevention of sexually transmitted diseases (STD) during follow-up [20].

Considering the complexity of this case, in which multiple comorbidities conspired to bring about an unfavorable outcome, our success was unexpected when we consider that both twins, although prematurely delivered, survived without major complications and the mother had an uneventful post-delivery outcome. These positive results came as a consequence of a multidisciplinary approach; however, the risks involved were so high that major efforts are to be taken by our medical community to prevent unplanned pregnancies in all patients with CF, especially when a serious comorbidity like the one in this case is present.

In Table 1, the comorbidities our patient presented during pregnancy are shown in bold.

**Table 1:** Risks described in the literature for each comorbidity

Risk attributed to each disease	Teen pregnancy	CF pregnancy	SLE pregnancy
	**Preterm birth**	**Preterm birth**	Preeclampsia
	**Low birth weight**	**Exacerbation**	Spontaneous abortion
	**Fewer months breastfeeding**	Requirement for mechanical ventilation	**Increased rate of flares**
	**Postpartum depression**	Gestational diabetes	Death
		Death	
		Infant with CF	
		Acute respiratory failure	
		Acute renal failure	

Bold indicates those present in our patient

## Data Availability

The patient’s data available in this paper are in the patient’s records in Hospital de Clínicas de Porto Alegre.

## Abbreviations

CF: Cystic fibrosis
SLE: Systemic lupus erythematosus
CFFPR: Cystic Fibrosis Foundation Patient Registry
ANA: Antinuclear antibody
VDRL: Venereal Disease Research Laboratory
FVC: Forced vital capacity
FEV1: First second of forced expiratory volume
ICU: Intensive care unit
NETs: Neutrophil extracellular traps
